# Spatial prediction of plant invasion using a hybrid of machine learning and geostatistical method

**DOI:** 10.1002/ece3.11605

**Published:** 2024-06-25

**Authors:** Liang Shen, Elizabeth LaRue, Songlin Fei, Hao Zhang

**Affiliations:** ^1^ Department of Statistics Qingdao University of Technology Qingdao China; ^2^ Department of Biological Sciences University of Texas at EI Paso EI Paso Texas USA; ^3^ Department of Forestry and Natural Resources Purdue University West Lafayette Indiana USA; ^4^ Department of Statistics and Probability Michigan State University East Lansing Michigan USA

**Keywords:** boosted regression tree, invasive plants cover, least absolute shrinkage and selection operator, ordinary kriging

## Abstract

Modeling ecological patterns and processes often involve large‐scale and complex high‐dimensional spatial data. Due to the nonlinearity and multicollinearity of ecological data, traditional geostatistical methods have faced great challenges in model accuracy. As machine learning has increased our ability to construct models on big data, the main focus of the study is to propose the use of statistical models that hybridize machine learning and spatial interpolation methods to cope with increasingly large‐scale and complex ecological data. Here, two machine learning algorithms, boosted regression tree (BRT) and least absolute shrinkage and selection operator (LASSO), were combined with ordinary kriging (OK) to model plant invasions across the eastern United States. The accuracies of the hybrid models and conventional models were evaluated by 10‐fold cross‐validation. Based on an invasive plants dataset of 15 ecoregions across the eastern United States, the results showed that the hybrid algorithms were significantly better at predicting plant invasion when compared to commonly used algorithms in terms of RMSE and paired‐samples *t*‐test (with the *p*‐value < .0001). Besides, the additional aspect of the combined algorithms is to have the ability to select influential variables associated with the establishment of invasive cover, which cannot be achieved by conventional geostatistics. Higher accuracy in the prediction of large‐scale biological invasions improves our understanding of the ecological conditions that lead to the establishment and spread of plants into novel habitats across spatial scales. The results demonstrate the effectiveness and robustness of the hybrid BRTOK and LASOK that can be used to analyze large‐scale and high‐dimensional spatial datasets, and it has offered an optional source of models for spatial interpolation of ecology properties. It will also provide a better basis for management decisions in early‐detection modeling of invasive species.

## Graphical abstract

Modeling ecological patterns and processes often involve large‐scale and complex high‐dimensional spatial data. Due to the nonlinearity and multicollinearity of ecological data, traditional geostatistical methods have faced great challenges in model accuracy. As machine learning has increased our ability to construct models on big data, we propose the use of new statistical models that hybridize machine learning and spatial interpolation methods to cope with increasingly large‐scale and complex ecological data.

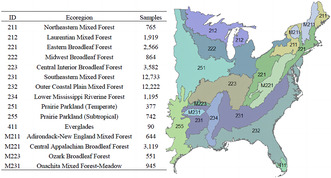

## 1 INTRODUCTION

With the development of Geographic Information System (GIS) and remote sensing technology, many large‐scale and complex spatial datasets are now available in the ecological domain. The growing availability of big data may have driven ecology into a new era of data mining‐based multidisciplinary science (Kanevski et al., 2009; Mccallen et al., 2019). Many researchers have paid attention to small‐scale studies of invasive plant ecology; however, small‐scale research hardly provides overall understanding of the complex biological invasions at macroscales (Pauchard & Shea, 2006; Soranno et al., 2014; Wiens, 1989). For example, attempts to resolve why a positive relationship is often found between species richness and invasion at large spatial scales versus a negative relationship at small spatial scales (Byers & Noonburg, 2003) require big ecological data (Iannone et al., 2016, 2018; Nunez‐Mir et al., 2019). Additionally, the accurate modeling of ecological patterns and processes that frequently vary across spatial scales has been challenging (Fei et al., 2016; Hamil et al., 2016; Pauchard & Shea, 2006), because of limitations in statistical models to handle complex ecological data that are often required to understand large‐scale ecology (Farley et al., 2018; Oswalt et al., 2015). Therefore, constructing efficient spatial statistical models to address the complex and high‐dimensional ecological data poses a challenge to many researchers (Conquest, 2008; Mccallen et al., 2019).

According to previous studies (Burrough & McDonnell, 1998; Du et al., 2020; Laslett et al., 1987; Li et al., 2011), current spatial interpolation models could be roughly categorized into four groups. (1) Deterministic interpolation models (inverse distance weighting [IDW], nearest neighbors [NN], trend surface analysis [TSA], etc.). Deterministic models have a wide range of applications because of simplicity (Collins & Bolstad, 1996; Isaaks & Srivastava, 1989; Mueller et al., 2004; Webster & Oliver, 2002). However, they are incapable of providing prediction variances. (2) Geostatistical models (ordinary kriging [OK], etc.). Geostatistics methods aim to construct a surface based on kriging techniques that use semi‐variograms for predicting continuous‐valued attributes (Burrough & McDonnell, 1998; Clarke & Farrow, 2001; Goovaerts, 1997; Mikhail, 2008; Pebesma, 2004). Geostatistical methods have proven to be powerful for predicting ecological distributions. However, as the sample becomes large, it is computationally challenging to invert the covariance matrix necessary for kriging. (3) Machine learning methods (Breiman, 2001; Cortes & Vapnik, 1995; Hsieh & Tang, 1998; Hsu et al., 1995; Schmidhuber, 2015) (e.g., support vector machines [SVM], random forest [RF], and deep learning [DL] algorithms). As the main technique in the big data and artificial intelligence era, machine learning can assist in applying ecological models across scales (Crisci et al., 2012; Gilardi & Bengio, 2009; Hung et al., 2017) for analyzing and predicting the spatial distribution of ecological patterns and processes. According to Du et al. (2020), machine learning has been applied as an important theoretic/technical support for addressing spatial data. Finally, (4) combined methods. Several types of combined methods have been proposed, such as linear regression combined with kriging (Hengl et al., 2004; Odeh et al., 1994), classification and regression tree combined with ordinary kriging (Erxleben et al., 2002), and support vector machine combined with inverse distancing weighted and ordinary kriging (Li et al., 2011). These models have been shown to be superior to the plain statistical methods in terms of predictive performance.

Based on the conclusion of Kanevski et al. (2008), an essential issue in spatial data analysis and modeling includes the exploration of adaptive data‐driven, multivariable, nonlinear, and robust models in very high‐dimensional spaces with good generalization ability. Such demands effectively often turn into the field of machine learning. Previous efforts to predict the large‐scale drivers of invasions have not taken advantage of combining the ability of machine learning with spatial interpolation models. To our knowledge, we were able to find only one study in ecology (Brus et al., 1996) that compared the performance of spatial interpolation models, among which the hybrid method (stratified ordinary kriging, StOK) performed the best in relation to the accuracy of the prediction.

To explore the large‐scale and complex ecological relationships from spatial data, we aimed to use the benefits of both machine learning and geostatistical modeling to construct hybrid models. In the present study, two hybrid machine learning interpolation algorithms, BRT residual ordinary kriging (BRTOK) and LASSO residual ordinary kriging (LASOK), were built for predicting plant invasion from the US Forest Inventory and Analysis (FIA) database. Here, we test the feasibility and performance of hybrid models to analyze the spatial correlations between many ecological features from a local to sub‐continental scale. We show that the hybrid of machine learning and spatial interpolation algorithms has great potential in analyzing high‐dimensional ecology data.

## 2 METHODOLOGY

### 2.1 Ordinary kriging

In spatial statistics, ordinary kriging (OK) is a linear interpolation method where the error variance is minimized (Burrough & McDonnell, 1998; Eldeiry & Garcia, 2010; Mikhail, 2008; Zhang & Wang, 2010). It assumes that the observations represent a partial realization of an underlying stochastic process that has a constant mean and finite variance. Kriging seems to be the linear unbiased prediction that minimizes the prediction variance. It therefore results in the best linear unbiased prediction (BLUP). Let $y\left(x_{i}\right)$ denote observation at location $x_{i}\in A$ (i=1,2,⋯,n), the kriging prediction at a new location $x_{0}$ is the weighted sum of the observations,

(1) $$\hat{y}\left(x_{0}\right)=\sum_{i=1}^{n}\lambda_{i}\cdot y\left(x_{i}\right)$$

where the coefficients $\lambda_{i}$ are determined by minimizing the prediction variance

(2) $$E{\left(y\left(x_{0}\right)-\sum_{i=1}^{n}\lambda_{i}\cdot y\left(x_{i}\right)\right)}^{2}$$

with the constraint,

(3) $$\sum_{i=1}^{n}\lambda_{i}=1$$

The constraint ensures that the predictor is unbiased. The solution to the minimization problem is given by a linear equation that involves either covariance function or variogram.

### 2.2 Boosted regression tree (BRT)

As an ensemble algorithm in machine learning, BRT combines regression trees (model the dependent and independent variable by recursive binary splitting) and a boosting (a technique to reduce both bias and variance) to improve the predictive performance of many simple and weak tree models (Carty et al., 2015; Elith et al., 2008). BRT algorithm has been widely used in the area of ecology.

BRT has an iterative procedure where tree‐based models are fitted iteratively by recursive binary partition to improve weak modeled observations until a minimum model deviance is obtained. The final BRT model actually is an additive regression model where all simple trees are multiplied by the learning rate based on all data. Thus, BRT can overcome the disadvantages of single tree models and fit complex nonlinear interaction relationships between variables.

### 2.3 Least absolute shrinkage and selection operator (LASSO)

LASSO was presented to develop the estimation precision and interpretability of regression models, and it was often used for variable selection in high‐dimensional data (Hastie et al., 2009; Tibshirani, 2011). As an extension of the ordinary least squares model, LASSO attempts to minimize the residual sum of squares with a penalty term. Consequently, it has a tendency to render many terms in the regression term to have zero coefficient, and this results in only a few terms or variables to be selected. Therefore, LASSO is particularly helpful to address the so‐called Small *n*, Large *p* problem (Hastie et al., 2009), which refers to data with the dimension of independent variables *p* larger than or comparable to the sample size *n*. Most statistical theory is based on asymptotic approximations that allow the sample size *n* to grow large (Lafferty & Wasserman, 2006). When the number of variables *p* in the model is large, however, this theory can be misleading. This is also known as the statistical *curse of dimensionality* (Wasserman & Lafferty, 2005), which refers to complexity of computation that arises when analyzing data in high‐dimensional spaces.

LASSO uses an $\ell^{1}$ constraint for both fitting and penalization of the model parameters in Equations (4) and (5), in which it penalizes the coefficients of regression variables by shrinking their corresponding coefficients to zero. After shrinking procedure, the variables that still have a nonzero coefficient are selected as part of the model (Hastie et al., 2009). Consider a dataset consisting of N samples, each of which contains p independent variables and a dependent variable. Let $y_{i}$ be the dependent variable and $x_{i}≔{\left(x_{1},x_{2},\cdots ,x_{p}\right)}^{T}$ be the vector of independent variables for the i−th sample. Then, the objective of LASSO is to solve

(4) $$\min_{\beta_{0},\beta }\left\{\frac{1}{N}\sum_{i=1}^{N}{\left(y_{i}-\beta_{0}-x_{i}^{T}\beta \right)}^{2}\right\}\;\text{subject to}\;\sum_{j=1}^{p}\left\|\beta_{j}\right\|\leq t$$

where βp=∑j=1pβjp1p is the standard $\ell^{p}$ norm, and t is the upper bound for the sum of the coefficients. This optimization process is identical to the parameter estimation that follows

(5) $$\hat{\beta }\left(\lambda \right)=\underset{\beta }{\text{argmin}}\left(\frac{1}{N}\sum_{i=1}^{N}{\left(y_{i}-\beta_{0}-x_{i}^{T}\beta \right)}^{2}+\lambda {\left\|\beta \right\|}_{1}\right)$$

Here, λ≥0 is a user‐defined parameter which controls the overall degree of penalty, the higher the value of λ, the greater power of the shrinkage.

### 2.4 The hybrid machine learning interpolation models

Due to the significant advantages of machine learning in handling complex high‐dimensional data, we proposed the hybrid of machine learning with spatial interpolation methods to cope with invasive plants cover data. Two hybrid machine learning interpolation models, the boosted regression tree residual ordinary kriging (BRTOK) and the least absolute shrinkage and selection operator residual ordinary kriging (LASOK), were constructed in this paper. Both BRTOK and LASOK construction algorithms followed a three‐step building structure:

1. BRT/LASSO was first used to fit the multiple variables to give the prediction,
2. then the residuals of BRT/LASSO (=*observed value − fitted value*) were derived at all sample locations, and OK was used to model local spatial correlation (Demyanov et al., 1998; Hengl et al., 2004, 2007; Li, 2013) at unvisited location, and
3. finally, the predicted value of invasive cover $\hat{y}\left(x_{0}\right)$ at an unvisited location $x_{0}$ was calculated by summarizing the fitted values and residual interpolation results:

(6)

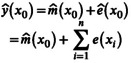

where $\hat{m}\left(x_{0}\right)$ is the fitted value of BRT/LASSO regression algorithm, $\hat{e}\left(x_{0}\right)$ is the estimated residuals at the unvisited location $x_{0}$, $e\left(x_{i}\right)$ is the residual of BRT/LASSO at location $x_{i}$, and $\lambda_{i}$ $\left(i=1,2,\cdots ,n\right)$ are the weights of OK decided by the spatial dependence structure of residual.

### 2.5 Cross‐validation and performance evaluation

Cross‐validation is a very useful tool for evaluating the performance of statistical models (Arlot & Celisse, 2010; Kohavi, 1995). It helps understand how the model can be generalizable to an independent dataset, and it is often applied to estimate model prediction precision. For *k*‐fold cross‐validation, the whole data are first randomly divided into *k* roughly equal‐sized groups, and then the model will be fit to *k*−1 groups (training data) and eventually calculates the error of the remaining group (testing data). The process is repeated *k* times so that each group can be used as a testing set.

In order to assess the forecasting precision of different algorithms, root mean squared error (RMSE) is often used as an error metric between predicted and observed numerical values (Belayneh et al., 2014; Hastie et al., 2009),

(7) $$\text{RMSE}=\sqrt{\frac{1}{N}\sum_{i=1}^{n}{\left(y_{i}-{\overset{⌢}{y}}_{i}\right)}^{2}}$$

where $y_{i}$ and ${\overset{⌢}{y}}_{i}$ represent the observed and predicted value, respectively. The smaller the value of RMSE, the closer the predicted value to the observed, thereby the higher the prediction accuracy.

In this paper, for assessing the performance of OK, BRT, BRTOK, and LASOK, RMSE was estimated by 10‐fold cross‐validation. Meanwhile, parameters optimizing for the above models were also selected by cross‐validation. Such an approach can be very time consuming if the volume of samples is large, and the dimensions of the data are very high. Therefore, it is necessary to restrict the range in which these values should be selected. In this study, the searching window size (*nMax*) in OK, BRTOK, and LASOK algorithms was selected in a range from 10 to 150; the number of trees (*nTrees*) in BRT and BRTOK method was determined in a range from 100 to 800; the *lambda* in LASOK algorithms was selected in a range from 0.01 to 1; and the Spherical model of variogram was selected to fit OK, BRTOK, and LASOK methods.

### 2.6 Software

All of the calculations were implemented in R version 3.5.1 environment (R Core Team, 2019). OK was performed with the gstat package of version 2.0‐6 (Bivand et al., 2008; Gr¨aler et al., 2016), BRT was executed with gbm package of version 2.1.8 (Ridgeway, 2007), BRTOK modeling was performed with gbm and gstat packages, and LASOK modeling was performed with the glmnet of version 4.0 and gstat packages (Friedman et al., 2010).

## 3 DATA AND STUDY AREA

### 3.1 Invasive plants data

The U.S. Forest Inventory and Analysis (FIA) program has been collecting invasive plant occurrence and distribution through all public and private US forests for several decades. It has provided large‐scale samples and high‐dimensional variables which can be used in statistical models to reflect local ecological differences of plots varying in environment and invasion of non‐native species (Cleland et al., 1997).

At the plot level, we obtained a series of 39 ecological variables that can be served as auxiliary information to improve the spatial prediction of invasive plant cover (Table 1). We obtained stand structural information (tree density and productivity), microhabitat (altitude, percent area forested), and diversity (species richness and phylogenetic diversity) of the tree communities based on FIA data from Iannone et al. (2016, 2018). Methods for these measurements can be found in Iannone et al. (2016, 2018). We obtained 17 climate variables from WorldClim Global Climate Data Version 1.4 (http://www.worldclim.org; Hijmans et al., 2005). We obtained an aridity index from Global Aridity Index (http://www.cgiar‐csi.org/data; Trabucco & Zomer, 2017) and soil carbon from the World Soil Information (http://www.isric.org; Batjes, 2015). All the 39 ecological variables were first standardized to have zero mean and unit variance, and for the sake of satisfying the normality requirement, log transformation was also applied to the dependent variable of invasive cover.

**TABLE 1:** Ecological features used to describe spatial variation in invasive plant covers.

Variable	Description	Data source
LAT	Latitude in decimal degree	Iannone et al. (2016)
LON	Longitude in decimal degree	Iannone et al. (2016)
Mean_Annual_Temp	Mean annual temperature (C × 100)	Iannone et al. (2016)
annual_Precip	Annual precipitation (mm)	Iannone et al. (2016)
Seasonability	SD of mean annual temp	Iannone et al. (2016)
Alt	Altitude in m	Iannone et al. (2016)
PLT_TPA	Trees/acre	Iannone et al. (2016)
Tpha	Trees/hectare	Iannone et al. (2016)
RelDen	0–1. reflects proportion of potential growth on the plot, or successional development	Iannone et al. (2016)
Prpfor	Proportion of plot that is forested	Iannone et al. (2016)
plt_drybio_adj	Aboveground dry‐wt biomass of native trees, English tons/acre	Iannone et al. (2016)
plt_drybio_ha	Aboveground dry‐wt biomass of native trees, English tons/hectare	Iannone et al. (2016)
native_spp	Native tree species richness	Iannone et al. (2016)
PD_all	Phylogenetic diversity of tree species	Iannone et al. (2016)
PSV_all	Phylogenetic tree species variability	Iannone et al. (2016)
PSV_all_var	Variance of phylogenetic tree species variability	Iannone et al. (2016)
PSR_all	Phylogenetic tree species richness	Iannone et al. (2016)
PSR_all_var	Variance of phylogenetic tree species richness	Iannone et al. (2016)
PSE_all	Phylogenetic tree species evenness	Iannone et al. (2016)
PSC_all	Phylogenetic tree species clustering	Iannone et al. (2016)
InvTotalCover	Sum of cover estimates for all invasive plants (can be greater than 100%)	Iannone et al. (2016)
Isotherm	Isothermality (Mean diurnal range / temperature annual range) × 100	https://www.worldclim.org/
maxtempwarm	Maximum temperature of warmest month	https://www.worldclim.org/
meandiurnrge	Mean diurnal range (mean of monthly [max temp − min temp])	https://www.worldclim.org/
meantempwetq	Mean temperature of wettest quarter	https://www.worldclim.org/
meantempdryq	Mean temperature of driest quarter	https://www.worldclim.org/
meantempwarm	Mean temperature of warmest quarter	https://www.worldclim.org/
meantempcold	Mean temperature of coldest quarter	https://www.worldclim.org/
mintempcold	Minimum temperature of coldest month	https://www.worldclim.org/
precipwetm	Precipitation of wettest month	https://www.worldclim.org/
precipdrym	Precipitation of driest month	https://www.worldclim.org/
precipseason	Precipitation seasonality (coefficient of variation)	https://www.worldclim.org/
precipwetqu	Precipitation of wettest quarter	https://www.worldclim.org/
precipdryqu	Precipitation of driest quarter	https://www.worldclim.org/
precipwarmqu	Precipitation of warmest quarter	https://www.worldclim.org/
precipcoldqu	Precipitation of coldest quarter	https://www.worldclim.org/
soilcarbon	Soil carbon in 0 to 20 cm depth	http://www.isric.org
tempanrge	Temperature annual range (warmest–coldest temperature)	https://www.worldclim.org/
tempseason	Temperature seasonality (standard deviation × 100)	https://www.worldclim.org/
Aridity	Aridity index (mean annual precipitation/mean annual potential evapotranspiration)	www.cgiar‐csi.org/data

### 3.2 Study area

**FIGURE 1:**
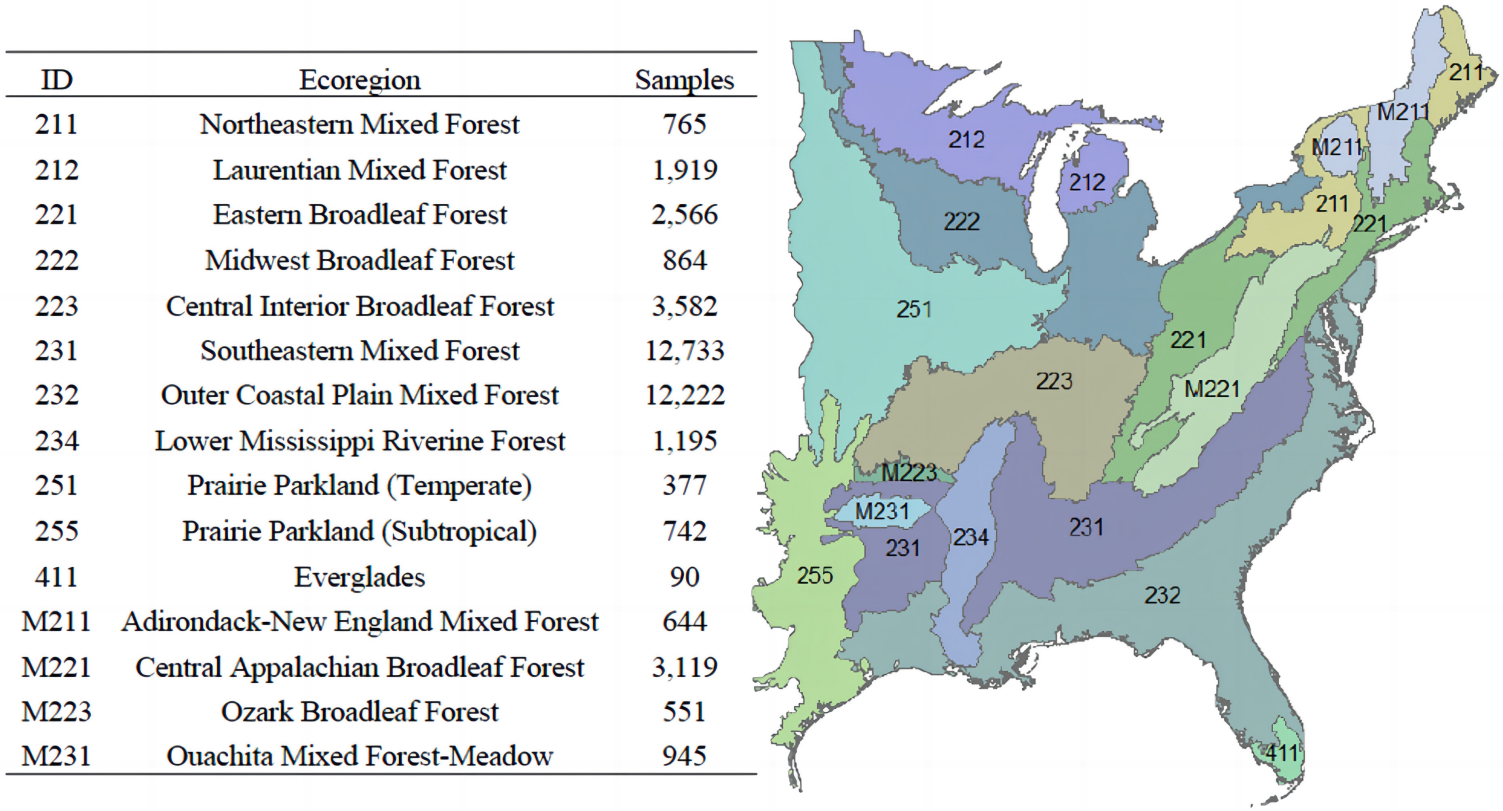
(left) Description of each ecoregion and its sample size; (right) Study area across the eastern United States, which was subdivided into 15 ecoregions as defined by Cleland et al. (2007) for the province level.

The homogeneous property of the FIA sampling design and its national sampling intensity of nearly one plot per 2400 hectares provides a unique opportunity to study plant invasions across time and space (Bechtold & Patterson, 2005). The study area was subdivided into 15 ecoregions following the province‐level delineation (hereafter called ecoregion) that is used by the US Forest Service (Cleland et al., 2007) (Figure 1, right) to incorporate the underlying variabilities such as environmental and geographic heterogeneity. Plots within a given section/division are more similar to one another with regard to a wide range of abiotic and biotic conditions than to plots within other ecoregions.

To test how the ecological conditions of recipient forest ecosystems were related to plant invasion, native tree and invasive plant data on 46,071 forested FIA plots in eastern USA (Iannone et al., 2016; Oswalt et al., 2012) were considered in this paper. After excluding plots with missing values, we eventually got 42,314 samples for analyses. Figure 1 (left) shows the number of samples that belong to each ecoregion.

## 4 RESULTS

### 4.1 Geostatistical analysis and prediction

Before analyzing the actual data of invasive plants cover, we first presented the spatial distribution maps of simulation study with a simple example from Burrough and McDonnell (1998), where only 155 observations were used to give the prediction at unknown locations (Figure S1a). In this simulation, five independent (or auxiliary) variables and one dependent (or target) variable were included and used to give the prediction in OK, BRT, BRTOK, and LASOK algorithms in the supporting information (Table S1, Figure S1).

For the real case, BRTOK and LASOK models were compared with OK and BRT methods using invasive plants datasets of 15 ecoregions in eastern USA (Figure 1). Statistics were computed for the most predictable composition of model parameters and model complexity in Table 2, where parameters with the minimum RMSE were selected as the optimal complexity. Regarding BRT, BRTOK, and LASOK, they all outperformed OK approach in all ecoregions except Prov_411. Results tabulated in Table 2 show that both BRTOK and LASOK overperformed OK in all ecoregions except Prov_411. Meanwhile, BRTOK performs better than BRT in all ecoregions, and LASOK also gives better predictions or is at least comparable to BRT.

**TABLE 2:** Optimal model parameter settings and prediction accuracies (by 10‐fold cross‐validation) of invasion cover by ecological variables across 15 ecoregions.

Ecoregions	Index	OK	BRT	BRTOK	LASOK
Prov_211	OP^a^	100	200	35/200	65/0.01
	RMSE	0.887	0.825	0.821	**0.820**
Prov_212	OP	70	100	70/100	70/0.02
	RMSE	0.655	0.660	0.653	**0.641**
Prov_221	OP	100	450	100/450	145/0.035
	RMSE	1.503	1.390	**1.386**	1.411
Prov_222	OP	100	100	90/100	130/0.02
	RMSE	1.233	1.203	1.197	**1.184**
Prov_223	OP	50	250	50/100	50/0.01
	RMSE	1.391	1.336	**1.319**	1.328
Prov_231	OP	55	600	60/200	55/0.01
	RMSE	1.452	1.381	**1.370**	1.394
Prov_232	OP	55	600	55/300	120/0.02
	RMSE	1.313	1.284	**1.277**	1.292
Prov_234	OP	95	100	30/100	105/0.025
	RMSE	1.333	1.312	1.300	**1.284**
Prov_251	OP	30	100	30/100	60/0.095
	RMSE	1.126	1.122	**1.117**	1.120
Prov_255	OP	55	100	50/100	55/0.01
	RMSE	1.173	1.183	1.181	**1.165**
Prov_411	OP	35	100	40/100	40/0.095
	RMSE	**1.329**	1.493	1.486	1.394
Prov_M211	OP	25	150	65/150	25/0.02
	RMSE	0.557	0.534	**0.532**	0.548
Prov_M221	OP	30	250	30/200	50/0.01
	RMSE	1.454	1.279	**1.275**	1.314
Prov_M223	OP	85	100	85/100	40/0.035
	RMSE	1.08	1.045	1.046	**1.045**
Prov_M231	OP	100	150	100/100	120/0.025
	RMSE	1.215	1.180	1.178	**1.163**

^a^ The optimal parameters (OP) of each algorithm are also shown in the table. For OK, the parameter is *nMax*; for BRT, the parameter is *nTrees*; for BRTOK, the parameters are *nMax* and *nTrees*; for LASOK, the parameters are *nMax* and *lambda*. The bolded value in each ecoregion shows the lowest RMSE values, which indicates the best accuracy among the four models.

**FIGURE 2:**
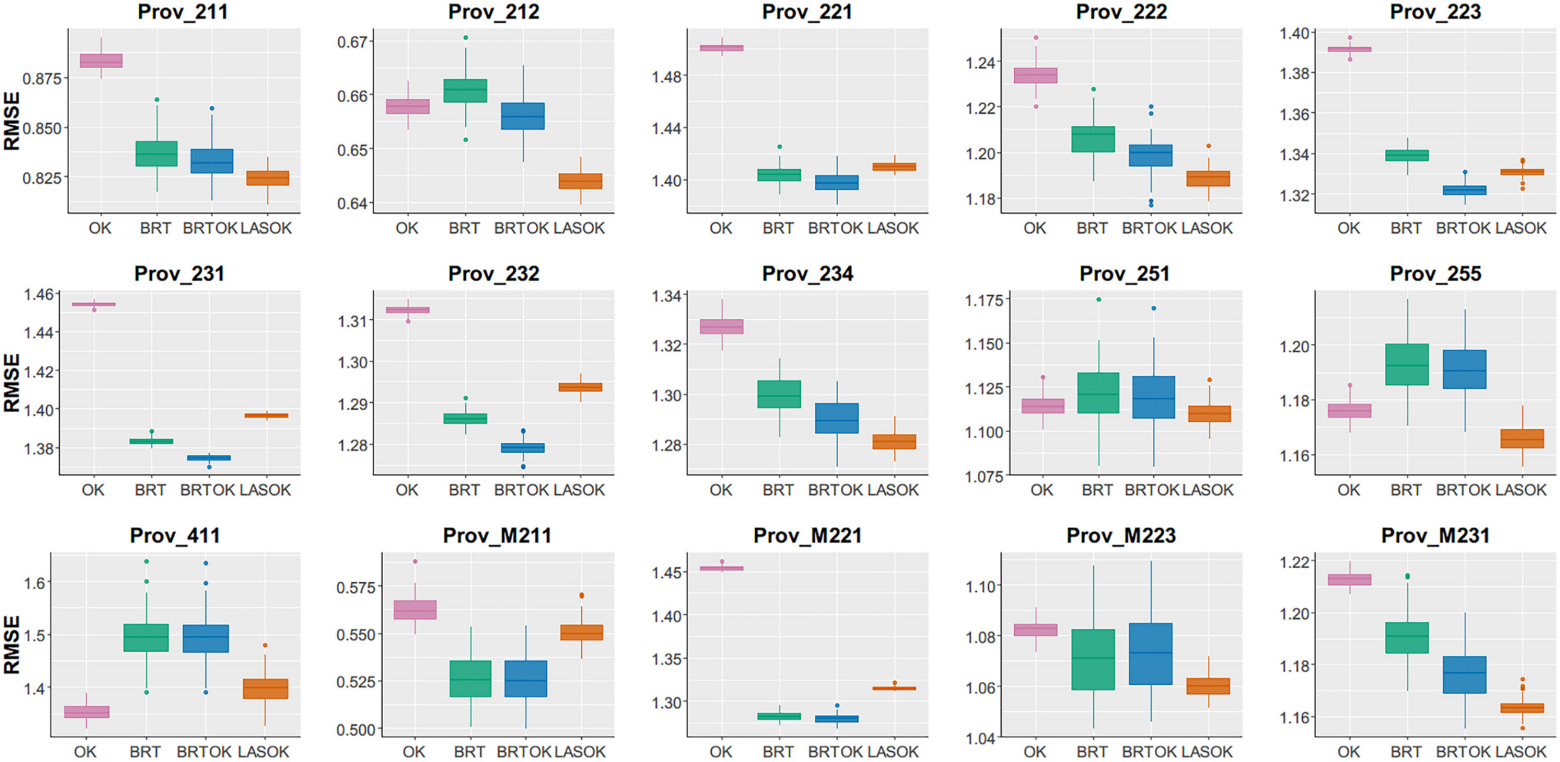
RMSE of OK, BRT, BRTOK, and LASOK for invasive plant cover: summary statistics are based on the 100 runs of the 10‐fold cross‐validation.

Additionally, in order to decrease the impact of randomness related to 10‐fold cross‐validation that each model may gain different portions of the data for prediction and validation, the learning process of 10‐fold cross‐validation was then repeated 100 times. The results of prediction error varied with the models in terms of RMSE (Figure 2). Clearly, LASOK was significantly more accurate than OK on the basis of paired‐samples *t*‐test (with the *p*‐value < .0001) in 14 out of 15 ecoregions, except for Prov_411. Simultaneously, BRTOK significantly outperformed the OK on the basis of the paired‐samples *t*‐test (with the *p*‐value < .0001) in 13 out of 15 ecoregions, except for Prov_255 and Prov_411. BRTOK was significantly more accurate in predicting invasion cover than BRT on the basis of the paired‐samples *t*‐test (with the *p*‐value < .0001) in all ecoregions. These results sufficiently demonstrated that the hybrid machine learning interpolation methods achieved better predictions than these existing common methods.

Furthermore, owing to the ability of BRTOK has to address big and noisy data, it performed the best in some ecoregions with large‐scale datasets (e.g., Prov_221, Prov_223, Prov_231, Prov_232, Prov_M211, and Prov_M221). Likewise, due to the ability of LASSO an $\ell^{1}$ constraint is used for both fitting and penalization of the model parameters, in which it penalizes the coefficients of regression variables by shrinking their corresponding coefficients to zero. Therefore, it can explain why LASOK performs the best among the four models in some ecoregions with relatively smaller datasets and similar dimensions (e.g., Prov_212, Prov_222, Prov_234, Prov_251, Prov_255, Prov_M223, and Prov_M231).

### 4.2 Selecting important variables

In addition to the good prediction ability of the two proposed machine learning interpolation methods, another application is that they have the capability of identifying important factors associated with the establishment of invasive cover, which can further provide more information about plant invasive cover.

**FIGURE 3:**
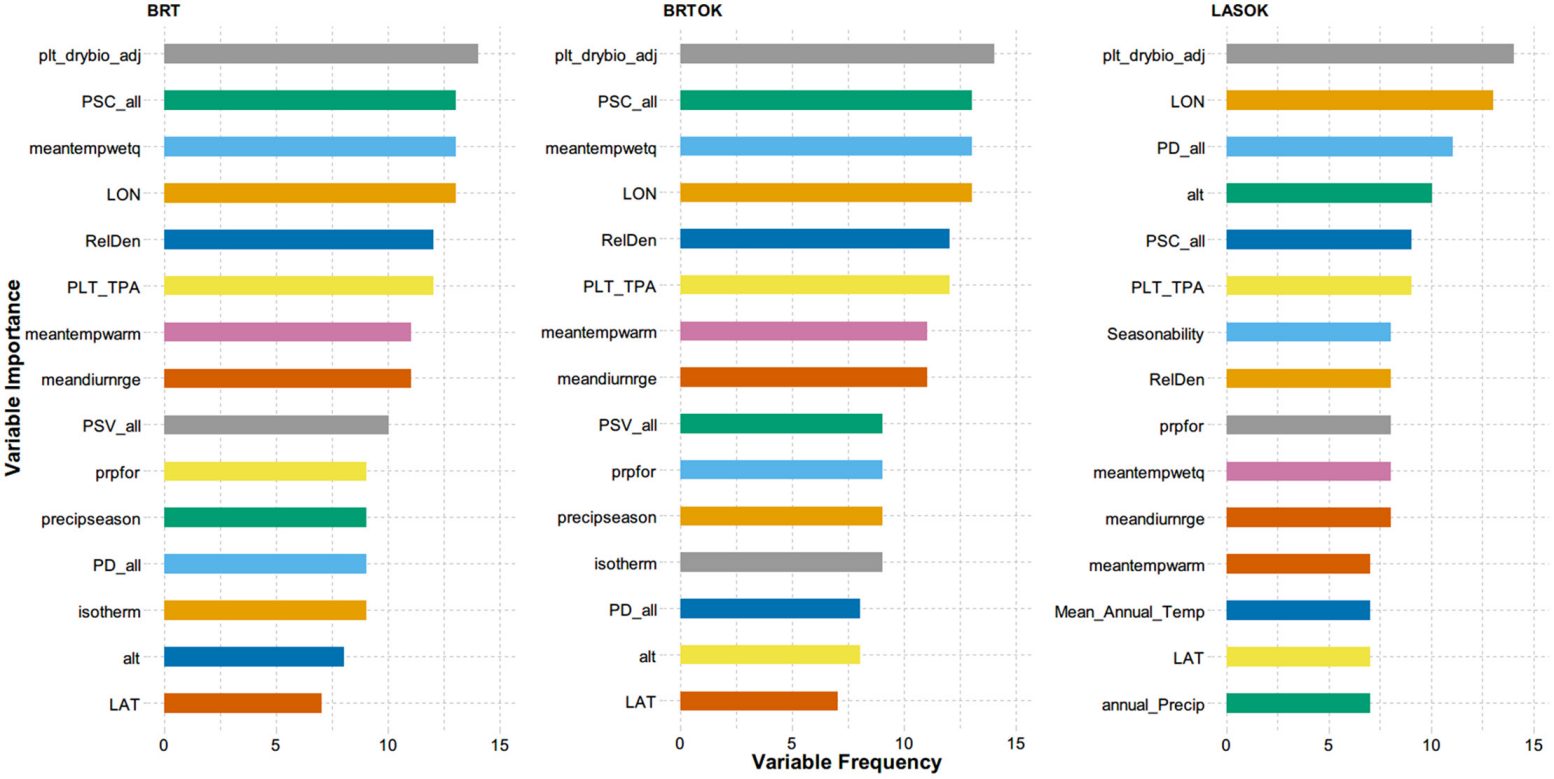
Relative importance of each variable as identified by BRT, BRTOK, and LASOK from 15 ecoregions that are associated with invasive plant cover (see Table 1 for ecological variable definition), which are shown in decreasing order.

According to Sections 2.2 and 2.3, LASSO can select important variables by shrinking some coefficients to zero. Meanwhile, variable importance of BRT is determined by averaging the relative importance of each variable, based on whether it was chosen to split during the regression trees building process. In this study, the frequency of the top 15 most important variables for BRT, BRTOK, and LASOK were ranked for all 15 ecoregions (Figure 3). In general, there are 12 common ecological variables chosen in the BRT, BRTOK, and LASOK models that are frequently associated with invasion cover.

Besides, in order to further test whether ecoregions were sensitive to the selected variables, an additional spatial predictions of invasive cover was given by removing four important variables (plt_drybio_adj, PSC_all, PLT_TPA, and PD_all). Take the ecoregion of Prov_211, for example, it was found that the predictive abilities of BRT, BRTOK, and LASOK perform worse than that in Table 1 after removing four important variables (Table S2).

## 5 DISCUSSION

The above results suggest that BRTOK and LASOK can successfully predict the general trend, but one needs to focus on the following three details in the utilization of these algorithms:

1. Prediction performance: Machine learning (ML) has become an outstanding technology because of its high precision and simplicity to apply (Liao et al., 2022). Incorporating ML models into invasive plants cover case may assist competent and precise ecological decision‐making. Thus, the aim of this study is to verify whether the hybrid of ML and traditional spatial interpolation algorithms could accurately predict the distribution of invasive plants cover and recognize important variables/predictors.
2. Application scope: The limitations of BRTOK and LASOK are that they are more complicated techniques that, if misused, may produce even poorer predictions than straightforward OK (Goovaerts, 1999). If the dataset is small, noisy, or non‐representative, then its probability distribution can be very far from the true probability distribution of the phenomenon. In addition, although machine learning methods can address ‘Small *n*, Large *p*’ problem, an important factor of the proposed hybrid of machine learning and geostatistical algorithms is the amount of samples needed to train the model. Normally, the more the number of training samples, the more accurate the model will get. According to Ott and Longnecker (2001), the minimum sample size (*n*) for training should be greater than 2*p* + 20, where *p* is the dimension of variables in the largest potential regression model. In this case, there are 39 variables in each ecoregion, so the minimum number of training samples is at least 98. Therefore, it can be explained why BRTOK and LASOK performed worse than OK in Prov_411 (Table 1, Figure 2), as the total sample size in Prov_411 is only 90. It means that when the amount of samples is extremely small (*n* < < 2*p* + 20), the hybrid machine learning interpolation method tends to fail, and using traditional geostatistical method may be a good choice. For these reasons, it can explain why the hybrid machine learning with spatial interpolation algorithms is more adapted to large datasets than to small ones.
3. Variable importance: The advent of GIS and remote sensing information technology has led to studies with complex and high‐dimensional variables. This will not only bring the curse of dimensionality causing high computation complexity (Wasserman & Lafferty, 2005) but also increase the possibility of correlation, multicollinearity, or redundancy among its variable/feature elements. Therefore, it is essential to select significant variables that associate with the target variable, especially for high‐dimensional settings in which some of the independent variables are strongly correlated with others or the dimensionality of independent variables is larger than sample sizes (Gusnanto et al., 2003; Maitra & Yan, 2008; Rodriguez, 2005; Shi & Tsai, 2010). Machine learning may help efficiently define subsets of variables in high‐dimensional data that relate to the outcome of interest. In particular, both BRT and LASSO have the function of obtaining the important variable; therefore, the proposed BRTOK and LASOK algorithms can help identify the important variables influencing the distribution of plant invasive cover. However, the correlation of the selected variables from this large‐scale dataset would require further experimental study to determine causation and cross‐scale relationships can complicate local or regional management when looking at a sub‐continental dataset. Hence, the variables selected by these algorithms merit further consideration for management potential of from a local to regional scale for invasive species.
4. Data source: One limitation of the data source is using some climate variables from version of Worldclim 1.4 rather than the latest version of Worldclim 2.0. As the main focus of the present study is to provide methodological improvement for ecological researchers, we will use the version of Worldclim 2.0 and its potential implications in the future study.

Technically, as machine learning models the mean of the response variables and essentially assumes independent identical distribution (i.i.d.) errors, ordinary kriging utilizes the second‐moment properties (i.e., covariance functions or semivariogram) under the assumption of constant mean. Therefore, the hybrid BRTOK and LASOK algorithms can sufficiently model the mean as well as utilize the spatial correlation for prediction.

## 6 CONCLUSIONS

Machine learning (ML) has made great success as an excellent prediction approach; it can recognize patterns in samples and construct models to predict attributes of the data with automatic computerized algorithms. Machine learning is especially appropriate for handling large‐scale data, analyzing the intricate relationship among various predictors, and easily incorporating new predictors into prediction models without any need of re‐adjusting the preprogrammed rules. Moreover, the process of feature selection can also be integrated into machine learning to assist recognize important predictors. Therefore, these characteristics make machine learning a powerful technology for leading to precise prediction in invasive plants cover.

In this study, two novel hybrid machine learning interpolation algorithms, BRTOK and LASOK, were designed for general spatial prediction of biological invasions. The main principle was to utilize the advantages of both machine learning and spatial interpolation methods, so that the proposed hybrid methods can not only extract useful information from large‐scale high‐dimensional dataset but also consider the trend estimation of the residuals. Over 15 ecoregions of invasive plant cover cases studied, the hybrid of machine learning and ordinary kriging algorithms were able to produce a modeled output of superior or at least comparable accuracy to commonly used models.

For large‐scale and high‐dimensional data in ecological science, the results indicate that both BRTOK and LASOK are preferred due to their advantages in handling big data. In practice, we recommend to apply both methods and choose one for the analysis, as they presented very similar performance and provided similar predicted values of invasive plant cover. When *n* is much smaller than 2*p* + 20, then OK is more recommended. More importantly, informative ecological features which may influence invasive plant cover can be further discovered by variable importance ranking with BRTOK and LASOK methods.

Based on the assessment results over 15 ecoregions, we demonstrated the effectiveness of BRTOK and LASOK that can be used to analyze large‐scale and high‐dimensional spatial datasets, and it has offered an optional source of models for spatial interpolation of ecology properties. We believe that the proposed hybrid machine learning interpolation models are promising tools in many application areas using large‐scale and high‐dimensional spatial data.

## AUTHOR CONTRIBUTIONS

**Liang Shen:** Methodology (equal); writing – review and editing (lead). **Elizabeth LaRue:** Data curation (supporting); formal analysis (supporting); writing – original draft (supporting); writing – review and editing (supporting). **Songlin Fei:** Conceptualization (lead); formal analysis (lead); methodology (supporting); supervision (lead); writing – review and editing (supporting). **Hao Zhang:** Funding acquisition (lead); methodology (lead); software (supporting); supervision (lead).

## CONFLICT OF INTEREST STATEMENT

The authors declare no conflicts of interest.

## Supporting information


Figure S1.

Appendix S1.

## Data Availability

The source data of this manuscript will be available in the Dryad Digital Repository. Sharing link: https://datadryad.org/stash/share/sVq2IrMuQOUriAOkO6fDatAarN5t8Iz9kSwZ83VQU_0.
