# Supplementary figures and images for: Spatial prediction of plant invasion using a hybrid of machine learning and geostatistical method
Source: Ecol Evol. 2024 Jun 25;14(6):e11605. doi: 10.1002/ece3.11605 (PMC11199124; doi:10.1002/ece3.11605)

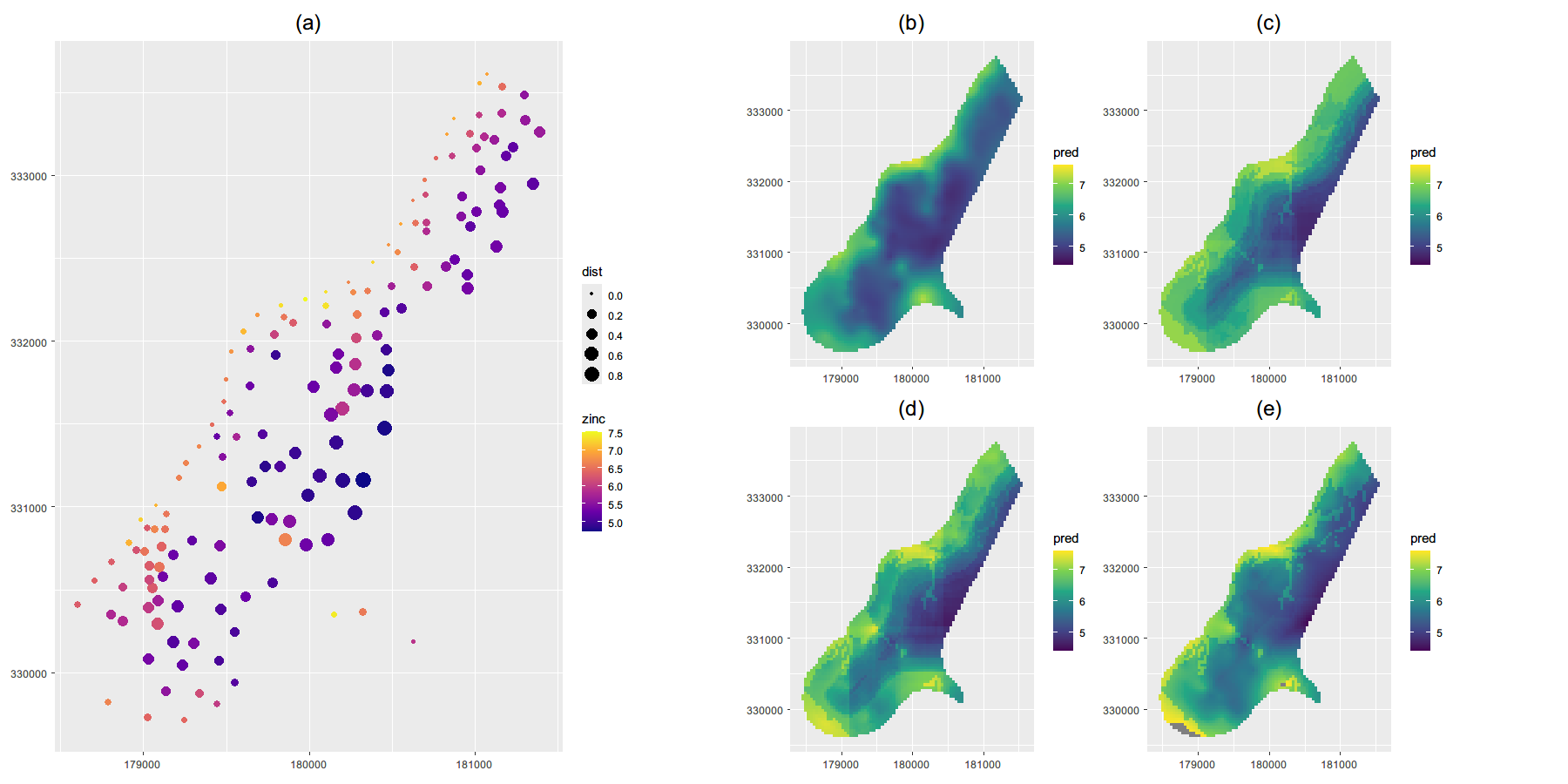

Supplement: Supplementary file 1 — Figure S1. [file ECE3-14-e11605-s002.tiff]
