# Supplementary material for: Spatial prediction of plant invasion using a hybrid of machine learning and geostatistical method
Source: Ecol Evol. 2024 Jun 25;14(6):e11605. doi: 10.1002/ece3.11605 (PMC11199124; doi:10.1002/ece3.11605)
Supplement: Supplementary file 2 — Appendix S1. [file ECE3-14-e11605-s001.docx]

**Spatial prediction of plant invasion using a hybrid of machine learning and geostatistical method**

**Supporting information**

**A simple simulation case study**

In this simulation, five independent (or auxiliary) variables and one dependent (or target) variable, *x*-coordinate (m), *y*-coordinate (m), distance to river Meuse (m), soil type, flooding frequency class, and topsoil zinc concentration (ppm), were included and used to give the prediction in OK, BRT, BRTOK and LASOK algorithms.

Table S1 lists the prediction results by 10-fold cross-validation obtained from the OK, BRT, BRTOK and LASOK models. It can be seen that the performance of LASOK and BRTOK are obvious better than that of OK and BRT.

**TABLE S1** Comparison of the four interpolation algorithms for accuracy of the prediction (RMSE).

| **Index** | **OK** | **BRT** | **BRTOK** | **LASOK** |
| --- | --- | --- | --- | --- |
| Optimal parameter | (*nMax*=)  25 | (*nTrees*=)  100 | (*nMax*/*nTrees*=)  100/100 | (*lambda*/*nTrees*=)  0.025/10 |
| RMSE | 0.401 | 0.325 | 0.318 | 0.317 |


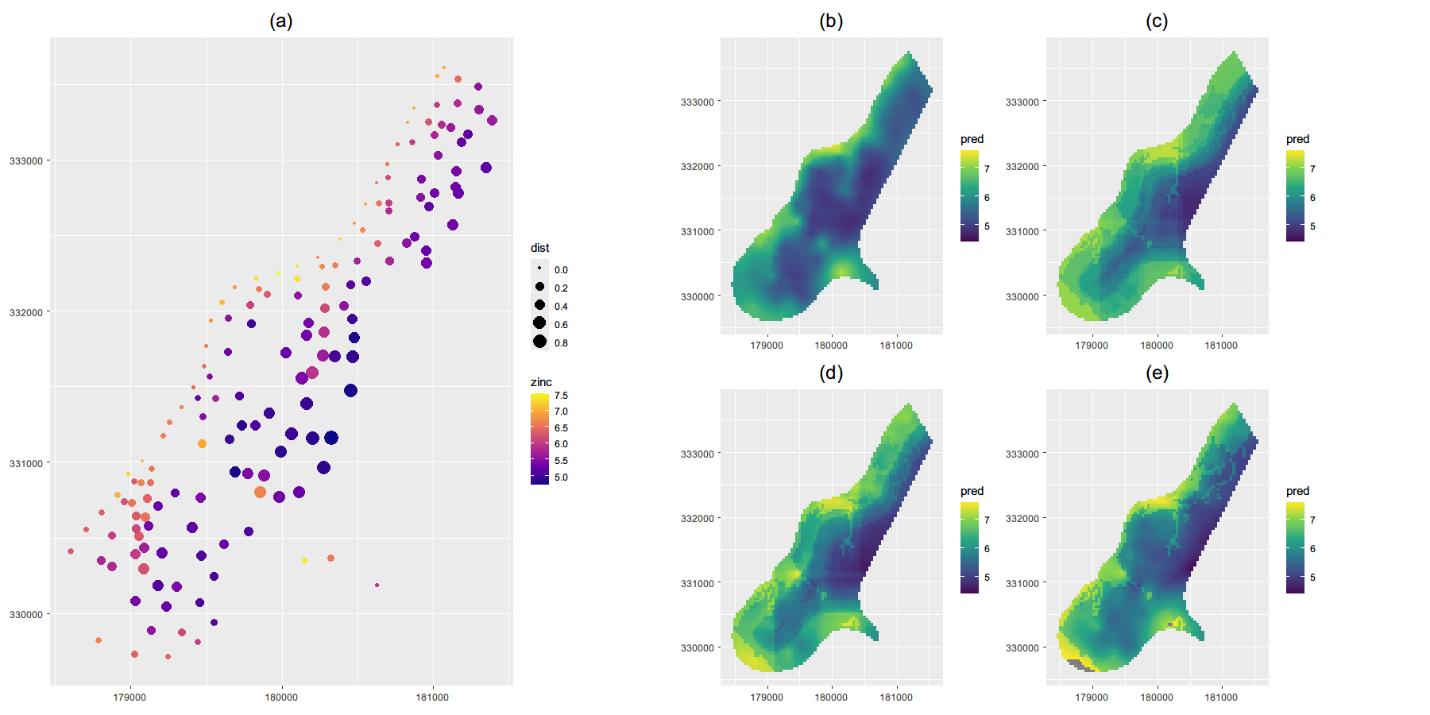


**FIGURE S1** A simple simulation example with only 155 measurements (Burrough and McDonnell, 1998; Pebesma, 2004). Comparison of spatial distribution maps of zinc pollution in topsoil with different models: (a) locations of 155 sampling units, (b) OK estimates, (c) BRT estimates, (d) BRTOK estimates, (e) LASOK estimates.

Comparison of different prediction models for mapping zinc pollutions with 155 measurements was shown in Figure S1, where the predicted results of OK estimates (Figure S1(b)) was different from that of BRT estimates (Figure S1(c)), BRTOK estimates (Figure S1(d)) and LASOK estimates (Figure S1(e)) over the entire spatial field. Clearly, BRTOK estimates (Figure S1(d)) and LASOK estimates (Figure S1(e)) yield more subtle details in the geospatial distribution of zinc pollution than OK map (Figure S1(b)).

**TABLE S2** Comparison of the four interpolation algorithms for accuracy of the prediction (RMSE) after removing four selected important variables in Prov_211.

| **Ecoregion** | **Index** | **OK** | **BRT** | **BRTOK** | **LASOK** |
| --- | --- | --- | --- | --- | --- |
| Prov_211 | Optimal parameter | (*nMax*=)  100 | (*nTrees*=)  100 | (*nMax*/*nTrees*=)  35/150 | (*lambda*/*nTrees*=)  115/0.01 |
|  | RMSE | 0.887 | 0.834 | 0.828 | 0.821 |
